# The scientific integrity of ADHD: A critical examination of the underpinning theoretical constructs

**DOI:** 10.3389/fpsyt.2022.1062484

**Published:** 2022-12-21

**Authors:** Sheelah Mills

**Affiliations:** Department of Health and Medical Science, Critical and Ethical Mental Health, Robinson Research Institute, University of Adelaide, Adelaide, SA, Australia

**Keywords:** DSM-III, philosophy of science, biological psychiatry, minimal brain dysfunction, cognitive psychology

## Abstract

Prior to the establishment and promotion of ADHD as a psychiatric disorder, the labels “minimal brain dysfunction” (MBD), “hyperactivity” (HA), and “learning disability” (LD) were diagnostic terms for children with hard-to-manage behaviors. At the time, these labels and the treatment interventions, especially the heavy reliance on stimulant medications, were subject to criticism. Nearly half a century later, these criticisms apply equally to ADHD, suggesting a disturbing lack of progress in this area of child psychiatry. Therefore, the aim of this article is to examine the scientific integrity of ADHD, to establish why this is the case. I use a philosophy of science framework to track the *initial thinking*, the *plausibilit*y, and the *acceptance* of ADHD. I establish that ADHD, along with the evolving biomedical model for psychiatry, was accepted in the third edition of the American Psychiatric Association's (APA) Diagnostic and Statistical Manual (DSM-III) as the result of bias and compromise between theorists' of different persuasions. Although initial ideas are expected to be subjective, they also need to demonstrate plausibility prior to empirical investigation. Research from the disciplines of biological psychiatry and cognitive psychology influenced the creation of ADHD, so I critically examine specific ideas that underpinned these disciplines at that time. I find these to be implausible and not congruent with current scientific knowledge, this extends to more recent theory. I conclude there is little good reason to consider DSM-III's concept of ADHD as empirically confirmed, nor do I find good reason to expect such confirmation will be forthcoming.

## Introduction

Attention-deficit/ hyperactivity disorder (ADHD) was originally called attention deficit disorder (ADD) when it first appeared in DSM-III (1). Inappropriate displays of inattention and impulsivity were listed as key symptoms indicative of a mental disorder. Seemingly, the new label encapsulated a variety of names that had previously been used to describe the condition, including: “Hyperkinetic Reaction of Childhood, Hyperkinetic Syndrome, Hyperactive Child Syndrome, Minimal Brain Damage, Minimal Brain Dysfunction, Minimal Cerebral Dysfunction, and Minor Cerebral Dysfunction [(1), p. 41]. A revised version of DSM-III [DSM-III-R, (2)] incorporated hyperactivity into the label, and ADD became ADHD. According to Faraone et al. (3) ADHD “occurs” in 5.9 % of youth and 2.5 % of adults, and is found throughout the developed and the developing world (p. 797). However, ADHD has long been associated with controversy and division (4).

Eric Taylor, Emeritus Professor of Child and Adolescent Psychiatry at King's College London, described how different views of the disorder caused division between psychiatrists and between other professionals, such as social workers and educators (4). Taylor discussed two extreme positions; at one end, ADHD is conceptualized as a biological condition of the brain, best treated with diet, drugs and behavior modification. Alternatively ADHD is conceptualized as a psychological variant, with problems resulting from “societal intolerance, based in emotional changes and requiring supportive and educational measures” (p. 69).

Such controversy and division is not new to psychiatry. Roger D Freeman, Professor of Psychiatry at the University of British Columbia, opened a lengthy article with the statement:

There is only one phrase for the state of the art and practice in the field of minimal brain dysfunction (MBD), hyperactivity (HA), and learning disability (LD) in children: a mess. There is no more polite term which would be realistic. The area is characterized by rarely challenged myths, ill-defined boundaries, and a strangely seductive attractiveness. These categories and their management, because of massive support from frustrated parents, professionals, government, and the drug and remedial-education industries, constitute an epidemic of alarming proportions—-but is the problem the disease or its treatment? [(5), p. 5]

Freeman concluded that there was no “epidemic” to justify the widespread use of stimulant drugs; rather, he considered this period in psychiatry to be “an unfortunate episode in the history of progressive medicalization of deviant or troublesome behavior” (p. 22). He outlined five reasons for his conclusions.

First, Freeman (5) argued that the ill-defined boundaries of the conditions labeled as MBD, HA, and LD did not distinguish between children failing to match society's expectations, at school, home, or elsewhere, from a small number of children whose difficulties were biologically based. This had permitted an unlimited expansion of diagnosis. Second, the ready availability of drugs meant they would continue to be overprescribed. Third, the vested interests of physicians and others in the field, no matter how well intended, when combined with the public acceptance of the “magic of science” (p. 22), had created a hard-to-resist vicious circle. Fourth, the MBD hypothesis had been blindly accepted by educators due to medical classifications being outside their area of expertise. Finally, Freeman argued that these diagnostic categories were supported by industry and by governments. Whereas, industry benefitted from the profits of manufacturing drugs, governments were provided with hope that deviance and antisocial behavior might be controlled.

Freeman (5) commented that the literature on the subjects of MBD, HA, and LD was too extensive to include an exhaustive review. He worried that “there is remarkably little well-established knowledge and a dearth of critical thinking which sometimes amounts to absurdity” (p. 6). Although he did not advocate a return to “preventable suffering” (p. 23), he argued that there was a need for research into current practices. The point about Freeman's article is that all of his criticisms of MBD, HA, and LD are equally applicable to ADHD 46 years later. This suggests an alarming lack of progress in this area of child psychiatry.

Freeman (5) doubted that the answer to why some people are different would ever be found. He considered the assumption that science would provide the tools to find these differences to be a “peculiarly Western ideology” (p. 23). But at the time of his writing, moves were already underway for psychiatry to change its approach to endorse this very ideology. The publication of DSM-III (1) is recognized as a seminal moment in psychiatry, because it signaled a change from a psychosocial model to a biomedical model (6). However, because of the interplay of scientific and political considerations between psychiatrists of different professional and therapeutic paradigms, this change occurred, not as the result of scientific endeavor, but more as the result of careful political maneuvering (7).

Bayer and Spitzer (7) argued that the political dimension of DSM-III was not unique to psychiatry, as research into the history and sociology of science found intraprofessional interests often influenced outcomes. In another article, specific to DSM-III's classification of psychiatric disorders in infancy, childhood, and adolescence, Spitzer and Cantwell (8) emphasized that the first step to developing diagnostic criteria, is when clinicians agree whether a description of a particular category seems, “on the face of it” (p. 350) to accurately reflect disorder. Although they did not name specific disorders, they noted that many of the new childhood categories were included in DSM-III with only face validity. They reasoned that the need for clinicians to communicate about the different types of disorders, meant they could not wait for a fully validated classification system. However, Spitzer and Cantwell cautioned that psychiatry had a responsibility to demonstrate other types of validity, otherwise there could be “no justification for continued use of those categories in a classification of mental disorders for general use” (p. 360).

As noted above (3), ADHD occurs in developed and developing nations at a prevalence rate of 5.9% of youth and 2.5% of adults. Correspondingly, the evidence of worldwide increases in stimulant dispensing (4, 9–13), indicates a wide acceptance of ADHD as a psychiatric disorder.

Nevertheless, by associating Freeman's (5) historical concerns with Taylor's (4) mention of controversy and division, I raised the question of whether knowledge about hard-to-manage behaviors has improved since ADHD appeared in DSM-III. Taking the position that ADHD was one of the disorders described by Spitzer and Cantwell (8) as having only face validity, I now ask whether other validation has occurred as intended, and if so, how. This question speaks directly to my use of the term scientific integrity, which is the expectation that the quality of theory and scientific processes, both prior to and post DSM-III, displays a standard of excellence to warrant ADHD remaining in DSM as a mental disorder.

In essence, the aim of this article is to hold psychiatry to account in a manner consistent with the philosophy of science (14). Traditionally, it is the role of the philosopher to address issues pertaining to the evidence and the justification of scientific claims, and although I am not trained in this discipline, I borrow a specific framework (15) to investigate the scientific integrity of ADHD and its place as a DSM classification.

### Kordig's distinctions as a framework for evaluation

Aufrecht (14) explained that in philosophy of science, issues about the evidence and justification of scientific claims are often dubbed “the *distinction between* “context of discovery” and “context of justification” (DJ or “context distinction”)” (p 151). Kordig (15) provided a standard account of what is meant by the discovery/justification (DJ) distinctions:

Logical empiricists distinguish discovery from justification. Discovery concerns the origin, creation, genesis, and invention of scientific hypotheses. Justification concerns their evaluation, test, defense, success, truth, and confirmation. … Discovery is subjective. Justification is objective. It is also normative. It determines what ought to be accepted. It evaluates scientific theories and hypothesis. Discovery concerns their origins. It may deal with the initial selection of facts for study. Justification asks whether the facts—-however selected—-constitute objective evidence for the hypotheses (p. 110).

Nevertheless, these distinctions attract controversy, because some scholars maintain that the two contexts are not clearly distinguishable (14, 16). Kordig (15) acknowledged the distinctions as ambiguous, but argued that much of the debate obscured the real issues of: “Are there good reasons for the discovery? Are they also good reasons for justification? Are they all inextricably linked to particular scientific theories?” (p. 110). He further claimed that there are three “proper” distinctions, which he called *initial thinking, plausibility*, and *acceptability*.

Kordig (15) described initial thinking with the words: *hit upon, think up, imagine*, and *make conjectures*. As the process of discovery is subjective, neither logic nor good reasons are essential to initial thinking. However, initial thinking is prior to, and psychologically distinct from, plausibility and acceptability. Plausibility may be expressed in various ways, but Kordig determined that a hypothesis needs to be plausible “*before* its test” (p. 115). Furthermore, Kordig warned that there is no guarantee that plausible lines of research will survive further testing.

Kordig (15) uses the term acceptability in line with the traditional justification distinction described above; it is when empirical investigation has found objective evidence to support the initial thinking. Acceptability is more stringent than plausibility, but the difference is one of degree. Good reasons support acceptability and “*prior to empirical test they also support plausibility*…” such good reasons need not, and usually do not, change when scientific theories change (p. 115–116). Good reasons help define regulative standards of excellence.

These regulative standards of excellence are consistent with my understanding of scientific integrity. Additionally, Kordig's (15) distinctions enable me to provide an account of the initial thinking, and the thinking behind that thinking. Together, they can be evaluated to show if there were good reasons for ADHD's entry into DSM-III. The plausibility distinction is particularly important, as it is this that determines a) the likelihood of ADHD symptoms being manifestations of a mental disorder, b) if empirical investigation will produce improved knowledge, and c) if there is good reason for ADHD to remain as a category in DSM.

### Structure of this evaluation

I divide this evaluation into three components, to reflect Kordig's (15) distinctions of initial thinking, plausibility, and acceptance. Section 2 provides the background details of the why and the how of the current biomedical model of psychiatry. I establish that there were good reasons for psychiatry to improve its diagnostic practices, but find the processes were political rather than scientific (7).

I then discuss this background in relation to the creation of ADHD. I detail the influence of Paul Wender, a prominent child psychiatrist and proponent of the diagnostic label of MBD. I link Wender's interest in attentional difficulties to research by cognitive psychologists.

As already stated, it is the plausibility of initial ideas that helps determine the likelihood of future empirical confirmation (15), hence section 3 is a description of the theoretical constructs associated with psychiatry's biomedical model and those that underpin cognitive psychology, including an early model of attention. These are then critiqued in section 4, and I conclude that the lack of plausibility undermines the scientific integrity of ADHD in its position as a DSM classification.

Despite this, ADHD continues to be diagnosed and treated as a psychiatric disorder, so the next section outlines the period following DSM-III, to ascertain reasons for this ongoing acceptance. One major contribution to more recent discourse came from the work of a lead expert, the cognitive psychologist Russell Barkley (17). My critique of Barkley's theory once again points to a lack of plausibility. I discuss how the theory contributed to claims of a link to frontalsubcortical abnormality. But now a recent publication indicates the claims were premature.

I then bring my discussion back to the concerns expressed decades ago by Freeman (5), where I argue that the problems he identified have not been satisfactorily resolved. Nor do I consider that psychiatry's creation of ADHD has improved knowledge about the how, or the why, of the behaviors deemed to be symptomatic of disorder. I conclude by suggesting future research and theory should avoid single cause explanations, and focus instead on the complex relationship between nature and nurture.

## The historical background of ADHD and its biological underpinnings

### Psychiatry's paradigmatic shift

Prior to the publication of DSM-III (1), psychiatrists' treatment approach for mental disturbances relied on a mixture of psychodynamic and psychoanalytic theory known as the psychosocial model. The emphasis of the psychosocial model was to understand an individual's ability to adjust to life stressors, and to explore personality, as well as childhood and parental influences. Additionally, psychiatrists looked for psychological forces that might be working at the subconscious level (18).

At the same time, historical accounts identified the 1970s as a time of crisis for psychiatry (6, 19). The rise of an intellectual group, known as the anti-psychiatrists, questioned how psychiatrists could consider themselves medical when the problems they were dealing with were mainly conceived as social (6). One prominent critic, Thomas Szasz, argued that mental illness was a “myth,” and that psychiatry was being used as a way to control nonconformists. Another critic, the influential philosopher Michael Foucault, opined that psychiatric classifications were the result of 18th century power relations (19).

There was, however, a more practical force at play, that of resource dollars. Insurance companies were disgruntled with the unregulated nature of clinician payments, and they questioned whether the then dominant psychosocial model demonstrated quantifiable effectiveness (19). Federal agencies also bemoaned a lack of clinical accountability, believing that poor standardization and fluid methods of assessment and treatment created a “bottomless pit” for resources and insurance dollars [(6), p. 403].

Dissatisfaction with psychiatry was also building from within the profession, especially among a group who considered the field's future lay within a biological framework (20). The success of lithium carbonate to stabilize moods created an awareness of the potential to develop psychiatric medicines to treat a wider variety of conditions (6). This in turn created an emphasis on a need for psychiatry to be more empirically based. For this to occur, explicit diagnostic criteria were required to gather homogenous research samples (6).

Finally, two specific events occurred to provide the catalyst for change (20). The first was the findings of the USA-UK Cross National Project released in 1971, which detailed a study that established discrepancies in hospital admissions between New York City and London. A lack of consistency in diagnosis between the two cities meant the symptoms of a patient in New York would produce a diagnosis of schizophrenia, but the same patient was likely to be treated for manic-depressive illness in London (20). These results, according to Shorter (20), meant the time had come for psychiatry to address the lack of reliability with regard to diagnosis. The second event identified by Shorter (20) and Wilson (6), was the impending revision of the International Classification of Diseases (ICD) by the World Health Organization (WHO). The WHO and the United States had an agreement that their respective models of disease classification should be revised simultaneously, this was to enable comparison of morbidity and mortality statistics, as well as to standardize diagnostic practices (6). To this end, Robert Spitzer was appointed in 1974 by the APA's President-Elect, to chair the Task Force of Nomenclature and Statistics to revise DSM-II (21). Mayes and Horwitz (19) commented that, although the intention was not for a revolutionary revision of DSM, Spitzer intended otherwise.

Spitzer was influenced by what came to be known as the Feighner criteria (6, 18, 20). Feighner et al. (22) presented criteria for 14 different categories of adult psychiatric illnesses. They described each category as having been “sufficiently validated by precise clinical description, follow-up and family studies to warrant their use in research as well as in clinical practice” [(22), p.57]. This specificity was at odds with analytical understanding of mental illness, but was in keeping with how Spitzer envisaged aligning DSM-III with the rest of medicine. Spitzer worked with the group at Washington University responsible for developing the Feighner criteria, to produce specific Research Diagnostic Criteria (RDC) to serve as a template for DSM-III adult disorders (6). No such template was provided to the Advisory Committee on Infancy, Childhood and Adolescence Disorders (23).

As chair of the task force, Spitzer was able to select committee members whose ideas were compatible with his own. Half of these members were in some way affiliated with Washington University (6). According to Wilson, the guiding principles for the task force were never debated, because their vision for the future direction of psychiatry was in place from the outset. It was their intention to apply medicine's disease model to psychiatric problems. The new manual was to be categorical, meaning a mental disorder was either absent or present, and clinical inference was to be kept to a minimum. Moreover, it would be an unequivocal rejection of psychoanalytical practices derived from Freudian theory (18).

Bayer and Spitzer (7) explained that the introduction of a categorical approach in DSM-III was initially met with strong resistance. Ultimately, and after a great deal of negotiation between the various forces about what should or should not appear in the manual, the Assembly of the District Branches met on May 12th 1979. A motion to accept DSM-III in its entirety was approved by an overwhelming majority, thus appearing to close the ranks of deep division. Bayer and Spitzer concluded that the process of achieving this settlement had more to do with politics than the “orderly pursuit of scientific knowledge” (p. 195). They argued that, because important extraprofessional interests were at stake, this should come as no surprise.

### The origins of ADHD

Freeman's (5) description of a mess is mirrored in historians' accounts of the period prior to the introduction of ADHD into DSM-III (24, 25). Psychodynamic processes were lengthy and expensive, and the search for underlying causes too often failed to provide remedies. This meant parents and teachers were left still having to deal with challenging behaviors. At the same time, the modification of hyperactive behavior with medications was becoming accepted with enthusiasm during the 1960s and 1970s, with it being increasingly endorsed in schools throughout the United States (5, 26). Pharmacological interventions, unlike psychotherapy, were easy to administer, brought immediate results, and were by comparison economically viable (25).

This use of pharmacological agents for troublesome behaviors suggests that the biomedical model was underway in child psychiatry before the publication of DSM-III. Rafalovich (24) described how “the nomenclature en vogue” (p. 405) prior to ADHD was MBD. The United States Public Health Service had officially adopted this label in 1966. In 1971, Paul Wender released a book called *Minimal Brain Dysfunction in Children*. According to Rafalovich, Wender wanted to unify a surfeit of different terms such as, minimal brain damage, minimal cerebral palsy, minimal cerebral dysfunction, maturational lag, and post-encephalitic disorder into the single clinical entity of MBD. Furthermore, Wender was opposed to the psychodynamic community's approach to childhood problems, because he saw them as only offering “band aid” solutions to complex problems [(24), p. 407].

Paul Wender was a member of DSM-III's Advisory Committee on Infancy, Childhood and Adolescence Disorders [1, p. xvii], which undertook the process of unifying various labels. But rather than the single clinical entity becoming MBD, it was named attention-deficit disorder (ADD) because:

The essential features are signs of developmentally inappropriate inattention and impulsivity. In the past a variety of names have been attached to this disorder, including: Hyperkinetic Reaction of Childhood, Hyperkinetic Syndrome, Hyperactive Child Syndrome, Minimal Brain Damage, Minimal Brain Dysfunction, Minimal Cerebral Dysfunction, and Minor Cerebral Dysfunction. In this manual Attention Deficit is the name given to this disorder, since attentional difficulties are prominent and virtually always present among children with these diagnoses [(1), p. 41].

From this explanation, especially the phrases “this disorder” and “these diagnoses”, it would appear that the prior terms listed above are either the same, or closely related to ADD.

Spitzer and Cantwell (8) reported on the basic features of DSM-III classification. They explained that ADD was presented in DSM-III as having two subtypes: “attention deficit disorder *with* hyperactivity and attention deficit disorder *without* hyperactivity” (p. 363). ADD with hyperactivity replaced DSM-II's (21) diagnosis of hyperkinetic reaction of childhood or adolescence, but there was no DSM-II equivalent for ADD without hyperactivity. Spitzer and Cantwell were unsure whether these subtypes represented two distinct disorders or two forms of the same disorder. They also reported on a “residual subtype”, for when some symptoms persisted in those with a previous diagnosis of a disorder. Terms such as MBD were suggested for this subtype, but were not adopted because they “are based on unproven assumptions of an organic etiology” (p. 363). Consequently only one type of ADD replaced a previous DSM-II classification, and no direction was given about the origins of the other subtype, nor the residual type.

According to Taylor (4), the new category of ADD in DSM-III replaced MBD, while retaining many of its “overtones” (p. 73). Taylor explained that the MBD acronym had originally stood for minimal brain *damage*, but many within the field argued against this definition, not least because a large epidemiological survey found no evidence to support the organic hypothesis. Although the word *dysfunction* had a different emphasis, it still attracted the same criticisms as the previous label, because, as Taylor pointed out, this nomenclature was rather “tendentious in assuming physical etiology” (p. 72).

Biological psychiatrists' solution to such criticisms was to find a definition based on “psychological changes rather than unknowable neurological ones” [(4), p. 72]. According to Taylor, the opportunity for such a definition came from studies of attention by academic psychologists, notably Roscoe Dykman and colleagues at the University of Arkansas, and Virginia Douglas and her team at McGill University. Apparently, Wender considered attention deficits “the key to understanding MBD” [(4), p. 73], and Dykman et al. had aligned their research into attentional difficulties to organically based deficits. In addition, this area of research was developing the tools for experimental investigation, which by Taylor's account, combined to set the stage for the creation of ADD.

Thus, it would appear that the idea of attention deficits solved several problems for the psychiatrists tasked with DSM-III classification. It provided a label that did not overtly implicate organic etiology, thus reducing the controversy of previous labels, there were tools already in place for future research, and according to Taylor (4). ADD was of great importance because, “it replaced the aetiological formulations of the past (which were unreliable because of the difficulty of assigning cases to causes) with a simple description of observable behaviors” (p. 73).

But these behaviors are also exceedingly common, as evidenced by data obtained from an epidemiological study conducted by Werry and Quay (27). In Urbana, Illinois, the teachers of children in the first three grades of school rated the prevalence of a variety of behaviors. The sample of 926 boys and 827 girls found 40.7% of ADHD-like behaviors among boys, and 17.65% among girls. Werry and Quay noted that “acting-out or disruptive and immature symptomology is almost uniformly more common in boys” (p. 5), with teachers perceiving boys to be more trouble than girls. The frequency of such behaviors from a non-clinical sample challenges the judgment of how they were deemed to be clinically relevant.

This point was made by Wilson (6) when he argued that the transformation of psychiatry following DSM-III led to a narrowing of the “psychiatric gaze”, with three interrelated consequences. The first, he identified as a loss of any concepts to do with the mind and consciousness, meaning residents are taught to focus on the “superficial and publically visible” (p. 408). The second was that a 45-min cross-sectional interview replaced lengthy evaluations, which had previously attempted to place a person's symptoms within the context of that person's developmental history. But to Wilson, the third and most important consequence of this narrowed gaze was that the simple descriptions, such as those praised by Taylor (4), “fosters the confusion of the easily observable with the clinically relevant” (p. 408), to the extent that a careful description of symptoms is now accepted as an adequate or even the proper assessment of a patient.

To summarize as per Kordig's (15) distinction of initial thinking; while there were good reasons for psychiatry to find a more reliable way to diagnose disorders, change was implemented through a political rather than a scientific process, and the current biomedical model was accepted as the result of committee agreement. Spitzer and Cantwell (8) were clear that some categories were included in DSM-III on the basis of face validity, with the expectation that other forms of validation would follow. But as these categories were formulated by like-minded committee members, it is possible that the plausibility of some of the ideas were not adequately challenged. Kordig argued that an initial idea needs to show good reason why it is plausible, and why it is likely to withstand empirical testing. In the next sections, I describe and evaluate the ideological underpinnings to which ADHD is inextricably linked. I demonstrate that DSM-III's acceptance of ADHD was not accompanied by plausible ideas, meaning the scientific integrity of the disorder was compromised at the outset.

## The ideological underpinnings of ADHD

### Biological psychiatry

Samuel Guze was a major player in the APA's move to a biomedical model. He contributed to the formulation of the Feighner criteria (22, 28), which were used to develop the Research Diagnostic Criteria (RDC) to guide the advisory committees to formulate the adult categories for DSM-III (23). Guze's (29) vision for psychiatry was outlined in an address to the Institute of Psychiatry at the Maudsley Hospital, London, where he pronounced: “*there is no such thing as a psychiatry that is too biological*. I say this even though I believe that we still know all too little about the physiology of the brain in most psychiatric conditions” [(29), p. 315].

Guze (29) expanded on his faith in biological psychiatry with a brief mention of how evolution had shaped the brain, “the organ of mental functions or what we call the mind” (p. 315). He then stated that the nucleotides making up the human genome programmed the brain, and they also determined our sensitivity to external stimuli. Moreover, Guze argued that these nucleotides are arranged “appropriately”, in such a way as to “shape the growth and development of the brain”. At the same time, the nucleotides provide the code that sets the limits to how brain cells “mature and survive” (p. 316). Guze's faith in molecular genetics was a prominent part of his rationale for biological psychiatry. He argued that the concepts and techniques of this field were opening up future possibilities, not only for understanding how genes work, but also for finding ways to manipulate them leading to endless opportunities for biology and medicine. Guze further hoped that such studies would put to rest, “the long-standing unfortunate debate within the field concerning the relative importance of nature and nurture” (p. 320).

This vision for the future of psychiatry meant that Guze considered himself “agnostic” about psychologically meaningful experiences. He was not convinced that any of “the usual range of human troubles” (p. 317) would be powerful enough to play a causal role in illness. Instead, he maintained that psychopathology occurred because the brain systems that mediated psychological functioning were failing. Once psychiatrists had identified the various organic abnormalities, treatment of these would ultimately lead to improved psychological functioning. These views are similar to those expressed by Paul Wender prior to the creation of ADHD.

### Wender's theory of MBD

Paul Wender (30) argued that the MBD syndrome was of considerable theoretical interest, because, “there is good reason for believing that it is frequently an antecedent of some of the common, more severe, and less well understood psychiatric disorders of later life” (p. 55). Wender noted that, historically, such disorders were thought to arise as an outcome of some sort of neurological damage. However, his view was that these psychiatric conditions were attributable to a metabolic dysfunction. He argued that this hypothesis “was given some weak support by the pharmacological agents which prove useful in the treatment of MBD” (p. 61). He considered the metabolic dysfunction to be a genetic expression, “probably in norepinephrine metabolism (although abnormalities or serotonin or dopamine metabolism cannot be ruled out)” (p. 69), and it was this dysfunctional metabolism that affected levels of arousal, subsequently diminishing the sensitivity of the “reinforcement system(s) of the brain” (p. 69).

In a later account, Wender (31) attributed two areas of dysfunction to MBD. These were behavioral problems and perceptual-cognitive problems. He pondered whether attentional difficulties were a characteristic of perception and cognitive difficulties, but his preference was for them to be viewed separately. He had found problems with attention present in “most if not all, MBD children” (p. 47). Another distinguishing feature identified by Wender was poor impulse control, which, together with academic underachievement, was one of the most common referring complaints. Wender's advice regarding diagnosis was that, in the absence of “absolute criteria” (p. 53), clinicians would need to decide whether to err on the side of “loose or stringent” criteria. He proposed that loose criteria would be more useful, because this would initiate a “therapeutic drug trial”, which he considered “unusually safe”. Wender was unconcerned that loose criteria might lead to overdiagnosis, with a few children being prescribed drugs unnecessarily, because to him, this was preferable to missing treatment due to stringent criteria.

Wender (31) had an extremely favorable view of the benefits of pharmacological interventions. He argued that stimulants, when effective, were “one of the most strikingly effective physical treatments in psychiatry, comparable, perhaps, only to electroconvulsive therapy in the treatment of serious endogenous depression” (p. 57). He did not consider the slowing down of the child to be the most remarkable outcome, as he noted other pharmacological agents have the same effect. His view was that stimulants and tricyclic antidepressants, while active, improve multiple areas of psychological functioning. Wender argued that aside from the noticeable increase in attention spans, the drugs also helped children to act in a more mature manner, cognitively, socially, and interpersonally.

Wender's (31) preference was for prescribing stimulants, because children often became tolerant of tricyclic antidepressants. He also discussed the use of antipsychotic drugs, but due to “occasional and idiosyncratic reactions” (p.58), he suggested they only be used in cases when children were extremely hostile or destructive. He advised that stimulants should be administered by increasing the dosage until an optimum treatment response was reached, or, if “side effects became objectionable” (p. 58). Interestingly for a drug Wender described as “unusually safe”, he also noted that the known adverse effects of stimulants included anorexia, insomnia, and increased nervousness.

This is only a brief and selective account of Wender's (30, 31) proposal. But the main points relevant to ADHD are: (a) Wender suggested that children diagnosed with MBD had a biochemical “lesion” that was an antecedent to adult psychiatric problems; (b) he was very clear about the role of medications for treating this hypothetical lesion; (c) he advocated loose criteria rather than stringent ones; (d) he did not consider the adverse effects of stimulants a problem; and finally because attentional problems were apparent in all children with MBD, he had a preference for them to be separated from other areas of cognition and perception.

Wender's theory of a metabolic failure within the brain system or systems, is in line with Guze's (29) vision for biological psychiatry. But if, as Taylor (4) asserted, it was research by psychologists that set the stage for ADHD, then it is necessary to provide details of psychology's understanding of attention.

### Psychology's conceptualization of attention

The Oxford Dictionary of Psychology describes attention thus:

**Attention** *n*. Sustained concentration on a specific stimulus, sensation, idea, thought, or activity, enabling one to use information-processing systems with limited capacity to handle vast amounts of information available from the sense organs and memory stores [(32), p. 62].

This description, especially the phrases, *information-processing systems, limited capacity*, and *memory stores* are specific to how cognitive psychologists attempt to understand the acquisition and use of knowledge (33).

Reed (33), the author of the undergraduate textbook C*ognition Theory and Applications*, described how this information-processing approach contrasted with the stimulus-response methods of behaviorism. He stated that behaviorists study observable behaviors and record people's responses to stimuli, but they do not consider the thought processes involved in the responses. By contrast, the information-processing approach attempts to understand what happens to the information between the stimulus and the response. Reed demonstrated this with a flow diagram consisting of a series of boxes and arrows, each box had a different word or phrase printed on it: *sensory store, filter, pattern recognition, selection, short-term memory, long-term memory* (p. 3). These are the stages of information-processing, and the arrows point to the direction between input and output.

Apparently cognitive psychologists devise ways to find out what occurs at each of these hypothesized stages (33). When a person has trouble performing a task, psychologists attempt to ascertain the specific stage that is the primary source of this difficulty. Reed credited the British psychologist Donald Broadbent with helping cognitive psychology's information-processing view of human cognition gain momentum, not least because Broadbent (34) used a *Mechanical Model of Attention* to demonstrate the role of attention in selective listening tasks.

According to Reed (33), Broadbent reasoned that people have difficulty listening to different messages played simultaneously into each ear, because “many sensory inputs can simultaneously enter the sensory store, but only a single input can enter the pattern recognition stage” (p. 5). From this, by Reed's account, Broadbent reasoned that attention is controlled by a filter, which he demonstrated with the use of a Y-shaped tube with a narrow stem. This stem was named the “limited-capacity perceptual channel”; and the two branches of the Y shape were considered to be the “sensory store”. The opening of the branches were the “ears”, which were wide enough for balls to be inserted. Each branch could hold several balls at a time, the idea being that the balls represented digits that participants in attentional studies were expected to recall.

At the junction between the branches and the trunk of the Y shape, there was a hinged flap. This was “the filter”, and it was designed to swing back and forth to allow balls from either side of the “sensory store” to enter. Reed (33) explained that the rationale to Broadbent's model, was that it takes time to switch attention, therefore the interval of time separating the insertion of balls was important. If the balls were timed to follow each other in one second or less, then the performance of the participant would deteriorate because the flap would not have time to switch back and forth. When the listener reported digits from one branch at a time the interval timing was not so important.

Although Broadbent's (34) model was designed to represent shifts in attention, it is an early example of how cognitive psychology models brain systems. Aside from the ideas of sensory stores, filters, pattern recognition, and so on, the appeal of such modeling seems to be associated with time, as models of this type are based on the assumption that information remains in storage for a finite amount of time. This appears to be one of the differences between the cognitive psychologists' approach and that of behaviorists (33). If an individual's timed performance deviates from his or her peers, especially if the differences are found to be “statistically significant”, then conclusions can be related to something being at fault within the internal information-processing stages, that is, the “mechanics” of the brain.

As to the actual term *cognitive psychology*, this was coined by Ulric Neisser in 1967, when he wrote a book with that title. Neisser (35) explained how this school of thought was influenced by the advent of computers:

Computers accept information, manipulate symbols, store items in “memory” and retrieve them again, classify inputs, recognize patterns, and so on. Whether they do these things just like people was less important than that they do them at all. The coming of the computer provided a much-needed reassurance that cognitive processes were real; that they could be studied and perhaps understood [(35), p. 5–6].

It is noteworthy that Taylor (4) commented that citations between Wender and psychologists such as Dykman and Douglas, did not contain the “more neurological writings of the past” (p. 73). As cognitive psychologists model the brain on a machine, rather than any contribution from neuroscience, this is not altogether surprising.

Thus it would appear, that regardless of how biological psychiatrists and cognitive psychologists attempt to understand abnormal behaviors, both schools of thought have an underlying assumption of specific “brain systems”. Moreover, these systems seem to be thought of as being pre-programmed and absolute (29, 33, 35). From this point of view, ADHD might be thought to occur because of a “glitch” in the programming of that person's brain. Therefore, and as per Kordig's (15) distinctions, the prospect of ADHD receiving full validation as a DSM category, has much to do with whether the ideas just described (29–31, 33) are plausible.

## The plausibility of the theoretical constructs underpinning ADHD

### Biological psychiatry

The views of Guze (29) and cognitive psychologists (33), both have an underlying assumption that the brain is largely a pre-programmed organ. Guze's (29) faith in biological psychiatry placed a heavy emphasis on the human genome, which together with his downplaying of psychologically meaningful experiences, pointed to a form of genetic determinism (36). According to Lerner and Overton, this is the idea, “that genes (in and of themselves) constitute the bedrock, essential causal agents for a wide range of living organisms” (p. 108). They explained that interactions between behavior and DNA methylation is bidirectional, and they emphasized: “*This point is key*. It underscores the absurdity of genetic reductionist models: *Genes do not determine behavior”* (p. 114). Genes alone do not work as a type of command center for human behavior, instead the role of biology across the course of development is founded on this bidirectional relationship between DNA methylation and behavior.

Another relevant point, relating to Guze's (29) reference about the “unfortunate” nature-nurture debate, is that, according to Lerner and Overton (36), epigenetics is an interactive nature-nurture concept. This means that separation of the two “through reductionist procedures” is not possible, therefore conclusions cannot be made about whether one or the other has a more important role in behavior and development (p. 115).

One of the main reasons that Guze (29) argued so strongly in favor of biological psychiatry, was because the study of biology had helped other areas of medicine to understand etiology and pathogenesis. From this, effective treatments had been developed, including providing physicians with opportunities to intervene early in disease processes. Therefore, the ultimate aim of Guze's vision was that psychiatry would deliver similar outcomes. The appeal of early intervention for later adult psychiatric conditions, was also part of the rationale for Wender's (30, 31) theory of MBD, and his support for psychopharmacology.

The APA (1) did not provide clear guidance as to why they considered attentional difficulties warranted entry into DSM-III, nor did they explain how they were able to combine a surfeit of previous labels into a single clinical entity. Taylor (4) commented that Wender “regarded attention deficits as the key to understanding MBD” (p. 73), presumably this “key” would also explain all the previous diagnostic labels that were united under the label ADHD. Interestingly, most of these descriptions used terms such as damage or dysfunction, but deficit implies that rather than something being broken, there is a shortage of something. Wender (30) discussed the monoamine theory and abnormalities relating to norepinephrine, serotonin, and dopamine. He argued that such abnormalities affected children's sensitivity to the “reinforcement system(s) of the brain” (p. 69). He also stated that his hypothesis was given weak support by the pharmacological agents used to treat MBD. Although Wender recognized this could not accurately determine etiology, he stated that the drug responsiveness “does suggest a fairly specific biochemical lesion” (p. 61). Therefore the intention at that time, might have had more to do with the idea of a chemical deficit impacting attention, rather than a deficit in attention per se. Such conjecture suggests an inextricable link between ADHD and the monoamine theory that was first proposed in the 1960s (36).

Ang et al. (37) discussed the monoamine theory, also known as the chemical imbalance theory, in relation to depression. The main premise was that some prescription drugs are able to target the basis of mood, initially noradrenaline was the focus of research, but serotonin was also considered relevant. When selective serotonin reuptake inhibitors (SSRIs) were introduced to the market in the 1980s, the monoamine theory gained further traction, both professionally and in the “popular psyche” (p. 2). Ang et al. explained how SSRIs were thought to inhibit the serotonin transporter proteins, thereby increasing the availability of synaptic serotonin. It was on this basis, that pharmaceutical companies promoted depression as being caused by a chemical imbalance, or more specifically a serotonin deficiency. Ang et al. trace debate and controversy in more recent times, but the relevant point is that “no consistence evidence of an association between markers of serotonin activity or concentration and depression has been found” (p. 4). In recent times some highly influential psychiatrists have attempted to distance the discipline from this explanation, claiming it to be both an “urban legend” and an “obsolete Kuhnian paradigm”, but it became widely accepted by the public and remains influential [(36), p. 1].

Although the monoamine theory, is associated with explanations for depression not ADHD, the finding that it is unsupported has implications for the reasons Wender considered attention deficits as “the key to understanding MBD” [(4), p. 73]. The key Wender was most likely hoping for, was evidence of a link between attentional difficulties and a metabolic dysfunction, and presumably he thought the experimental tools developed by psychologists would help provide this evidence. However, the likelihood of such an occurrence hinges on the plausibility of the information-processing conceptualization of attention described earlier.

### Cognitive psychology

In my account of the origins of ADHD, I described the link between psychiatry and cognitive psychology, including how Wender “regarded attention deficits as the key to understanding MBD” [(4), p. 73]. I discussed how this helped set the stage for the creation of ADHD, and how Taylor considered this new label important because it replaced previous aetiological formulations with a “simple description of observable behaviors” (p. 72). Aside from my comments about the behaviors being extremely common, there is the additional puzzle of trying to understand why, at the dawn of biological psychiatry, a disorder was created by relying on a school of thought that models brain processes on the workings of a computer, thereby ignoring “the structure and the mode of development of the nervous system” (38).

The brain does not function in the precise manner of a machine. Nor is it “programmed” by the human genome and developed by “appropriately” arranged nucleotides as per Guze's (29) description above. Multiple factors contribute to its development, including, and most importantly, individual differences (38–42). Details of the works of Edelman and Fuster are beyond the scope of this article, but a pertinent point made by Edelman was that:

One of the main organizing principles … is that each brain has uniquely marked in it the consequences of a developmental history and an experiential history. The individual variability that ensues is not just noise or error … it is an essential element governing the ability of the brain to match unforeseeable patterns that might arise in the future of a behaving animal. No machine we are familiar with incorporates such individual diversity as a central feature of its design [(39), p. 71].

Similarly, Fuster (42) explained it is universally accepted that memory and learning are the result of the modulation of synaptic transmission. Neurons are interconnected and groups of neurons are excited by stimuli occurring at the same time, this leads to stronger connections between the synapses. But Fuster's point that supports Edelman's view of individual experience, is that Fuster postulated that “all cognitive representations, that is, all items of memory and knowledge consist of networks of cortical neurons that have been associated by experience, whether that is the experience of the species … or the experience of the individual organism” (p. 126). In short: experience counts, there is no one size fits all blueprint for programming the brain as per the suggestions of the theoretical constructs described above.

Edelman (38) considered much of cognitive psychology's theorizing to be a source of embarrassment, especially the representation of memory. He pointed out that as it is us, humans, who invented the computer, and the algorithms designed to store and retrieve information, cognitivists need to account for exactly how a human brain can be programmed in such a manner. The brain is not a simple input-output machine, and for this reason, Edelman considered cognitive psychology's representation of the brain an “intellectual swindle” (p. 229), one that is “global and endemic”. Moreover, Edelman argued that the cognitive enterprise was a “scientific deviation”, the critical errors of which are as “as unperceived by most cognitive scientists as relativity was before Einstein and heliocentrism was before Copernicus” (p. 14). He further argued that the cognitivist approach rested on a structure that was “incoherent and not borne out by the facts” (p. 14).

Fuster (42) also bemoaned inaccurate representations of cognition, including from within his own discipline:

However, by theoretically extrapolating the evidence from primary areas to upper associative areas, and by over-interpreting some behavioral results of cortical lesions, it has been incorrectly assumed that not only complex sensory features or movements, but also *specific cognitive functions*, are represented in associative areas. Thus some neuroscientists have been led to believe that there are cortical modules or “centers” for perception, memory, language, attention, and executive control, among other cognitive functions. And thus a more or less academically condoned “neophrenology” has emerged (p. 125–6).

Another noteworthy criticism came from within cognitive psychology during the era that Wender turned his attention to this discipline. Ulric Neisser, whose contribution to this school of thought was considerable (33), expressed concern that many of the experimental techniques devised, were seemingly developed for the sole purpose of recording “precise timings of stimuli or responses while avoiding the necessity of introspection altogether” [(35), p. 6]. Neisser argued that it was unlikely that any satisfactory theory of human cognition would be established by experiments that provide inexperienced subjects, “with brief opportunities to perform novel and meaningless tasks” (p. 8). Yet this description befits Broadbent's mechanical model, which Reed (33) credited for helping the information-processing view of human cognition to gain momentum.

Altogether it is apparent that the mid-20th century ideas about genetics and the brain that underpinned the creation of ADHD are not congruent with current knowledge. Guze's vision is suggestive of genetic determinism (36); Wender's idea of a chemical lesion has not been supported empirically, and is now described as an obsolete Kuhnian paradigm, or an urban legend (37); and cognitive psychology's information-processing model has been described by neuroscientists as an intellectual swindle (38), and a form of neophrenology (42). Such a lack of plausibility indicates that ADHD was accepted in DSM, without first demonstrating good reason to expect eventual empirical confirmation.

Thus, far I have provided the historical context prior to DSM-III, including the reasons for psychiatry's move to a biomedical model, and the thinking underpinning this change. I have evaluated specific theoretical constructs and found them unsupported by current understanding and knowledge. All of this belongs to what Taylor (4) described as the prehistory of ADHD, but Taylor argued that the story should not be allowed to end at this point. On this basis, I provide a brief overview of an influential post DSM-III theory, which, despite belonging to the same school of psychology critiqued above, has been widely cited, thus contributing to an ongoing acceptance of ADHD. Not, however, as per Wender's original idea of a chemical lesion, but more as a frontalsubcortical abnormality.

## ADHD post DSM-III

Since 1980 DSM-III has been revised and updated three times (2, 43, 44). In DSM-III and DSM-IV (43), ADHD was originally listed in the overall diagnostic category of: *Disorders usually first diagnosed in infancy, childhood and adolescence*. The latest edition, DSM-5 (44), placed ADHD into a newly developed category, *Neurodevelopmental disorders*. Given this shift in categorization, it is reasonable to expect that a transparent scientific process would have occurred, one which reflects an improved understanding of the relationship between the symptoms and a central nervous system disturbance or disturbances.

This also suggests as per Kordig's (15) distinctions, and Spitzer and Cantwell's (8) expectations, that sufficient objective evidence now exists to demonstrate good reasons for accepting ADHD as a valid psychiatric disorder, one that has earned its place as a DSM classification. This challenges my assertion that Freeman's (5) decades old concerns remain relevant and indicative of a lack of progress in this area of child psychiatry. However, I now argue that it was not objective evidence that contributed to keeping the idea of ADHD alive; instead it was the actions of those with vested interests, partially aided by a theory proposed by a leading authority on ADHD, Russell Barkley.

### Russell Barkley and his theory of ADHD

Barkley's (17) theory was published by the high-impact peer-reviewed journal the *Psychological Bulletin*, thereby indicating that it was deemed credible and scientifically sound. As of 12/09/2022, a search in the database Scopus for the term “ADHD” returned 39,484 documents. Barkley's theory has the greatest number of citations, with 4,996. It is currently influential, because it was cited 275 times in 2021 and has 185 citations listed for 2022.

Barkley (17) developed this theory of ADHD because, not too surprisingly given the above criticisms, it was apparent by the mid-1990s that researchers were unable to confirm the notion of an attention deficit. For this reason, and because, according to Barkley, research on ADHD was “nearly atheoretical” (p. 66), he put forward a theory he described interchangeably as a “model”. His explanation is too lengthy to review here, however, a point relevant to the plausibility of ADHD, is that Barkley's field is psychology and this theory is an information-processing model of the type critiqued above.

The central hypothesis to Barkley's (17) theory is that the signs and symptoms of ADHD are not due to a deficit in attention, but rather a deficit of *behavioral inhibition*, which interferes with the ability to delay responding. Despite behavioral inhibition being key, Barkley's explanation is confusing, making it difficult to understand the where, what, and how of this purported deficiency, although it seems to be some sort of “thing” that exists independently somewhere in the prefrontal cortex.

In an effort to gain insights into Barkley's background knowledge, I reviewed his supporting literature, and found that behavioral inhibition is consistent with descriptions of impatient/impulsive behavior, also recognized as poor self-control (17). Scrutiny of his evidence found only 12 of the studies cited had anything to do with impulsivity, and they were all reminiscent of Edelman's (38) “intellectual swindle”, Fuster's (42) “neophrenology”, and Neisser's (35) criticisms. All described procedures where children performed “novel and meaningless tasks” (p. 8) in a “laboratory” away from their regular learning environments. The tasks do not seem to have been standardized, nor did the studies build on each other to provide a comprehensive account of knowledge at that time. The ages across these studies ranged from 2.6 years (45, 46) to 21.7 years (47), suggesting that developmental differences were not taken into account. The number of participants per study ranged from 19 (48) to 90 (47), the combined number of participants from the 12 studies totaled 459. As to the actual data, these were created by recording the timing of the subjects' performances on the aforementioned meaningless tasks. The average differences between ADHD children and controls were calculated and found to be statistically significant, thus providing evidence of poor self-control. But perhaps it is the manner by which Barkley built his theory that is the most baffling aspect of this work.

Barkley's (17) central claim was that poor behavioral inhibition impacted all aspects of “executive functioning”, which he argued could be divided into four separate components, the identification of which came from an essay by Jacob Bronowski (49). This was an interesting and puzzling choice, because Bronowski's aim was to explain why human and animal languages evolved differently. There is no knowledge relevant to ADHD as the essay has nothing to do with the disciplines of biology, neurology, psychology, psychiatry, or developmental science. Furthermore, the essay is part of a posthumous collection of Bronowski's previously unpublished writings, and as it was unedited, there is a general lack of clarity to his central argument.

Barkley (17) linked this model of executive functioning to Fuster's (40) theory about the prefrontal lobes. He did this because, by his account, Bronowski “attributed the four executive functions to the prefrontal lobes” [(49), p. 70], Bronowski (49) did not refer to executive functions nor the prefrontal lobes. Moreover, Barkley ignored most of Fuster's key points, including a detailed and informative theory of how the brain processes information. This was quite unlike the computer based model of cognitive psychology. It was described as a “perception-action cycle” (p. 177), and it clearly demonstrated the interaction between the senses, behavior and the environment. Neither the senses, the environment nor perception featured in Barkley's theory. Although borrowing from other disciplines is not necessarily a problem, Barkley's proposal rested almost entirely on an essay that Bronowski (49) wrote to throw some light on his special interests “namely the language of science and the language of poetry” (p. 104).

Maybe to many academics, particularly those with links to industry funding, this unlikely source was unimportant because the theory opened new avenues of research into ADHD. Barkley (17) concluded by stating that research should seek to identify “the degree to which stimulant medication differentially affects the domains of executive functions and motor control in ADHD” (p. 85). As he had listed 29 different behaviors as “subfunctions” of these domains, this provided multiple opportunities for broad experimentation with medications.

When Barkley wrote the theory (17), he was a professor at the Department of Psychiatry and Neurology, University of Massachusetts Medical Centre, and the theory was funded by grants from the National Institute of Mental Health (NIMH) and the National Institute of Child Health and Human Development. Consequently, it might have seemed reasonable to have expected a high standard of scholarship from a leader in the field, especially one with decades of experience. This expectation might have seemed further justified, when Joseph Biederman, Chief of the Clinical and Research Programs in Pediatric Psychopharmacology and Adult ADHD at the Massachusetts General Hospital, and Stephen Faraone, a Distinguished Professor in the Departments of Psychiatry and Neuroscience & Physiology at SUNY Upstate Medical University, referenced Barkley, at least in part, to assert that ADHD was due to “frontalsubcortical” abnormalities (50, 51).

Faraone (50) and Biederman (51) stated that neuroimaging studies had found structural and functional pathological changes to support their claims of frontalsubcortical abnormalities. They cautioned that neither these changes, nor the genetic variants associated with ADHD, were sufficiently distinct for a diagnosis. Nevertheless, they considered neurobiological data to be useful for providing insights into cause and pathophysiology, but acknowledged that more work needed to be done (51). But now, despite decades of research, the neurobiological data remains insufficient to provide objective evidence of frontalsubcortical abnormalities (3).

### Current knowledge of ADHD

The World Federation of ADHD (3) released an update to an original International Consensus Statement on ADHD (52). The purpose of the update was to catalog “important scientific discoveries” (p. 792) from the last 20 years, based on a review of 208 evidence-based statements from high quality meta-analyses and very large studies. Faraone et al. claimed that this review enabled them to make firm statements about “the nature, course, outcome causes, and treatments that are useful for reducing misconceptions and stigma” (p. 792).

As to the neurobiology of ADHD, Faraone et al. (3) posed the question of: “What have we learned from studying the brains of people with ADHD?” (p. 796). To answer this they described two classes of research, the first was performance on psychological tests that “study mental processes”, and the second was direct examination with neuroimaging studies. Multiple studies were listed, but the conclusion was the same as the Biederman and Faraone's (51) earlier study, that of only “typically small” (p. 797) differences that were insufficient for diagnosing disorder. This conclusion was supported with a reference to Thome et al. (53).

The Thome et al. (53) study had nothing to do with neuroimaging studies, and may have been included in error, nevertheless it further weakens the biological view of ADHD. Thome et al. were commissioned by the World Federation of ADHD and the World Federation of Societies of Biological Psychiatry (WFSBP) to identify the phenotypic characterization of ADHD. The criteria for the Thome et al. study were derived from those developed for Alzheimer's disease (AD), which included validation in neuropathologically confirmed AD cases. However, Thome et al. advised that, with ADHD, finding an ideal biomarker “is hampered by the fact that the fundamental feature of ADHD neuropathology is elusive and post-mortem validation is hardly [sic] to achieve” (p. 381). The candidate biomarkers reviewed by Thome et al. were extensive, but they concluded that “none of the risk genes identified so far exhibits a sufficiently robust effect in order to fulfill the definition criteria of a true ADHD biomarker” (p. 389).

Apart from the Thome et al. (53) study not being a relevant citation for conclusions about brain imaging studies, it is curious that is not mentioned anywhere else in the Faraone et al. (3) consensus update despite it being an important scientific discovery. Twenty years prior, in an annual report for Johnson and Johnson (J&J), Biederman and Faraone, in their respective roles of director and co-director for the Johnson & Johnson Center for Pediatric Psychopathology at the Massachusetts General Hospital, noted the precariousness of ADHD's medical status. They stated that genetic, brain imaging, and epidemiological studies were needed to demonstrate the validity of ADHD (54). They recognized that without such data many clinicians “question the wisdom of aggressively treating children with medications, especially those like neuroleptics, which expose children to potentially serious adverse events” (p. 3). Furthermore, they were concerned that parents, patients and clinicians were exposed to a media that frequently questioned the legitimacy of childhood disorders.

Negative exposure by the media was part of the rationale for the first International Consensus Statement on ADHD (52), to which Biederman and Faraone were signatories. The statement was badged as “a reference on the scientific findings concerning the disorder” (p. 89), which apparently was needed to refute inaccurate media reports that portrayed ADHD as a myth or benign condition. Barkley et al. stated that comparing the arguments of a handful of non-expert doctors to mainstream scientific views, gave the impression that there was considerable disagreement over whether ADHD was a real medical condition. They insisted that no such disagreement existed. They further insisted that “numerous studies” had linked ADHD to several specific areas of the brain, notably “the frontal lobe, its connection to the basal ganglia, and their relationship to central aspects of the cerebellum” (p. 90). They claimed that the genetic contribution to the disorder was the highest for any psychiatric disorder, with one specific gene being “reliably” associated with ADHD. At the time there were 12 different scientific teams working to identify more. The tone of the statement was hubristic, especially the insistence that questioning the validity of ADHD “is tantamount to declaring the earth flat, the laws of gravity debatable, and the periodic table in chemistry a fraud” [(52), p. 90].

These claims by Barkley et al. (52) were not the same as the concerns expressed in the J&J report (54), nor is there any such certainty in the updated consensus statement (3). Although the stated reason for the Faraone et al. update was to report on purported important scientific discoveries, there was no direct claim of validation. Instead, in its place they painted “a picture of the disorder”:

ADHD is a chronic disorder in which developmentally inappropriate symptoms of inattention and/or hyperactivity/impulsivity lead to impairments in many aspects of living. … There are multiple genetic and environmental risk factors that accumulate in various combinations to cause ADHD. These risk factors lead to subtle changes in multiple brain networks and in the cognitive, motivational, and emotional processes they control [(3), p. 806].

The disorder still hinges on the value judgment of “inappropriate”, but now it is the symptoms that lead to impairment, rather than being signs of an impairment. The vague reference to genetic and environmental influences accumulating to cause ADHD, speaks to Lerner and Overton's (36) discussion about the bidirectional interaction between genes and the environment, and to Edelman (38, 39) and Fuster's (40–42) arguments about the role of experience in brain development. It also speaks to a seldom recognized area of research, the question of whether the environment of a child deemed to be “abnormal” is equal to one not so considered. Or, as Freeman (5) asked, “is the problem the disease or its treatment?” (p. 5).

Lerner and Overton (36) argued that such bi-directionality made it impossible to separate the roles of nature and nurture to explain development and behavior. This means that the alternative view identified by Taylor (4), that of ADHD being a psychological variant arising out of societal intolerance, is also weak due to the reliance on a specific aspect of nurture. Nevertheless, the Faraone et al. (3) picture is quite different from that painted by Barkley et al. (52), perhaps because it has become increasingly clear that the mid-20th century ideas that underpinned the origins of ADHD are untenable.

This does not mean that the behaviors associated with ADHD are not challenging, but by Kordig's (15) distinctions, a lack of objective evidence means there is no good reason to accept that ADHD has moved beyond face validity as described by Spitzer and Cantwell, (8). Moreover as the ideological underpinnings discussed above have not fared well in my critical evaluation, there is no good reason to believe objective evidence will eventuate.

## Summary and conclusion

Of the five reasons, Freeman (5) gave to support his use of the word “mess”, the first was to do with ill-defined boundaries, which he argued did not clearly differentiate between biological and societal problems. When ADHD was created as a new clinical entity for DSM-III, it was accompanied by symptom checklists, which provide the boundary between ADHD being present or absent. However, data collected by Werry and Quay (27) indicated that these symptoms are exceedingly common in early school years. I linked this to Wender's preference for loose criteria, and I cited Wilson's (6) concern that DSM-III had created the confusion that careful description of the easily observable makes them clinically relevant. Altogether, there is little evidence that DSM symptom lists have resolved the boundary problem identified by Freeman, if anything they have added to it, as the employment of such loose criteria has always pointed to a strong likelihood of overdiagnosis.

The second, third and fifth points raised by Freeman (5), those of the availability of drugs, vested interests, and industry profits, are all closely connected. The drugs remain readily available, and recent decades have witnessed an ever-increasing upward trajectory in their use worldwide (4, 9–13), indicating that drug companies invested in ADHD are making healthy profits. Although space precludes a discussion of competing interests, a list at the end of The World Federation of ADHD International Consensus Statement (3) is testament to the extent of pharmaceutical involvement. Similarly, a look at Barkley's website (russellbarkley.org), shows the large amount of intellectual property he owns as an outcome of his standing as an expert.

Freeman's (5) hard-to-resist vicious circle between vested interests and the public acceptance of the magic of science, speaks to the core of biological psychiatry, as evidenced by Guze's (29) address and Wender's (30, 31) theorizing. The thinking underpinning the biological model, especially with Guze, seemed to be that, because medicine has gained much from studying applied biology, and because psychiatry is a medical specialty, the same science would work for psychiatry. In the case of ADHD, Wender turned to psychology because he thought they had developed the tools for working science's “magic”. But as, psychology's methodology was critiqued by Edelman (38) because it ignored the biology of the brain, this is somewhat problematic for a discipline that describes itself as “biological”.

As to Freeman's (5) fourth point, that of educators blindly accepting medical classifications because they are outside their area of expertise, this extends to all lay people. Such acceptance is based on trust precisely because of a lack of expertise, with this trust comes an expectation of rigorous scientific processes and ethical behavior. Ethical behavior means providing accurate information about the nature of psychiatry's classifications, to do otherwise compromises the scientific integrity, not only of ADHD, but also of psychiatry's professional standing.

In conclusion, the use of Kordig's (15) distinctions enabled me to investigate ADHD from its genesis through to current times, thereby providing support for my argument that the problems identified by Freeman (5) have not been resolved. Freeman pondered if science had the necessary tools for finding answers to why some people are different, the creators of ADHD thought they had identified such tools, but my analysis has found this not to be the case. Furthermore, the attempts to reduce hard-to-manage behaviors down to molecular genetics, chemical imbalances, or attention deficits, has not improved knowledge or understanding about hyperactive, impulsive, and inattentive behaviors. However, unlike Freeman, I do believe that science has the tools to better understand these behaviors, but any future approach needs to keep in mind Lerner and Overton's (36) argument that nature and nurture are not mutually exclusive. Single cause explanations should be avoided in favor of any approach that takes multiple levels of functionality into account.

## Author contributions

The author confirms being the sole contributor of this work and has approved it for publication.

## References

[1] American Psychiatric Association. Diagnostic and Statistical Manual of Mental Disorders. 3rd ed. Washington DC: American Psychiatric Association (1980).

[2] American Psychiatric Association. Diagnostic and Statistical Manual of Mental Disorders. 3rd rev ed. Washington, DC: American Psychiatric Association(1987).

[3] Faraone SV Banaschewski T Coghill D Zheng Y Biederman J Bellgrove MA. The World Federation of ADHD International Consensus Statement: 208 evidence-based conclusions about the disorder. Neurosci Biobehav Rev. (2021) 128:789–818. 10.1016/j.neubiorev.2021.01.02 33549739 PMC8328933

[4] Taylor E. Antecedents of ADHD: a historical account of diagnostic concepts. ADHD Atten Defic Hyperact Disord. (2011) 3:69–75. 10.1007/s12402-010-0051-x 21431827

[5] Freeman RD. Minimal brain dysfunction, hyperactivity, and learning disorders: Epidemic or episode? School Rev. (1976) 85:5–30. 10.1086/443311

[6] Wilson M. DSM-III and the transformation of American psychiatry: a history. Am J Psychiatry. (1993) 150:399–410. 10.1176/ajp.150.3.399 8434655

[7] Bayer R Spitzer RL. Neurosis, psychodynamics, and DSM-III: a history of the controversy. Arch Gen Psychiatry. (1985) 42:187–96. 10.1001/archpsyc.1985.01790250081011 3883941

[8] Spitzer RL Cantwell DP. The DSM-III classification of the psychiatric disorders of infancy, childhood, and adolescence. J Am Acad Child Psychiatry. (1980) 19:356–70. 10.1016/S0002-7138(09)61059-1 6157706

[9] Hollingworth SA Nissen LM Stathis SS Siskind DJ Varghese JM Scott JG. Australian national trends in stimulant dispensing: 2002–2009. Aust N Z J Psychiatry. (2011) 45:332–6. 10.3109/00048674.2010.543413 21184644

[10] Klau J Bernardo CDO Gonzalez-Chica DA Raven M Jureidini J. Trends in prescription of psychotropic medications to children and adolescents in Australian primary care from 2011 to 2018. Aus N Z J Psychiatry. (2021) 00048674211067720. 10.1177/00048674211067720 34963342

[11] Thoutenhoofd ED. New techniques of difference: on data as school pupils. Stud Philosophy Educ. (2017) 36:517–32. 10.1007/s11217-016-9528-1

[12] Saiz LC. Pay attention to the Attention Deficit Hyperactive Disorder (ADHD): Between an uncertain nature and a hyperactive prescription. Drug Therapeutics Bull Navarre Spain. (2013) 21:1–18. Available online at: http://www.navarra.es/home_en/Temas/Portal+de+la+Salud/Profesionales/Documentacion+y+publicaciones/Publicaciones+tematicas/Medicamento/BIT/Vol+21/DTB+Vol+21+n+5.htm (accessed December 1, 2022).

[13] Stern HP Lipman J Andersen SL Bossaer JB Thigpen J. Risks of stimulant use for attention deficit hyperactivity disorder on the developing brain: Primum non nocere. Clin Pediatr. (2017) 56:805–10. 10.1177/0009922817706148 28459152

[14] Aufrecht M. Reichenbach falls—and rises? Reconstructing the discovery/justification distinction. Int Stud Philosophy Sci. (2017) 31:151–76. 10.1080/02698595.2018.1424760

[15] Kordig CR. Discovery and justification. Philos Sci. (1978) 45:110–7. 10.1086/288782

[16] Hoyningen-Huene P. Context of discovery and context of justification. Stud History Philosophy Sci A. (1987) 18:501–15. 10.1016/0039-3681(87)90005-717365444

[17] Barkley RA. Behavioral inhibition, sustained attention, and executive functions: constructing a unifying theory of ADHD. Psychol Bull. (1997) 121:65–94. 10.1037/0033-2909.121.1.65 9000892

[18] Horwitz AV Grob GN. The troubled history of psychiatry's quest for specificity. J Health Polit Policy Law. (2016) 41:521–39. 10.1215/03616878-3620797 27127255

[19] Mayes R Horwitz AV. DSM-III and the revolution in the classification of mental illness. J Hist Behav Sci. (2005) 41:249–67. 10.1002/jhbs.20103 15981242

[20] Shorter E. The history of nosology and the rise of the diagnostic and statistical manual of mental disorders. Dialogues Clin Neurosci. (2015) 17:59–67. 10.31887/DCNS.2015.17.1/eshorter 25987864 PMC4421901

[21] American Psychiatric Association. Diagnostic and Statistical Manual of Mental Disorders. 2nd ed. Washington, DC: American Psychiatric Association (1968).

[22] Feighner JP Robins E Guze SB Woodruff RA Winokur G Munoz R. Diagnostic criteria for use in psychiatric research. Arch General Psychiatry. (1972) 26:57–63. 10.1001/archpsyc.1972.01750190059011 5009428

[23] Cantwell DP. DSM-III studies. In: Rutter M, Tuma AH, Lann IS, editors. Assessment and Diagnosis in Child Psychopathology. London: David Fulton (1988). p. 3–36.

[24] Rafalovich A. Psychodynamic and neurological perspectives on ADHD: exploring strategies for defining a phenomenon. J Theory Soc Behav. (2002) 31:397–418. 10.1111/1468-5914.00167

[25] Smith M. Roy Porter student essay prize winner: hyperactivity and the evolution of American psychiatry, 1957–1980. Soc History Med. (2008) 21:541–59. 10.1093/shm/hkn060

[26] Lee RM. Psychopharmacology and hyperactivity: the imperfect panacea. J Clin Child Adol Psychol. (1981) 10:90–2. 10.1080/15374418109533022

[27] Werry JS Quay HC. The prevalence of behavior symptoms in younger elementary school children. Am J Orthopsychiatry. (1971) 41:136–43. 10.1111/j.1939-0025.1971.tb01114.x 5539486

[28] Robins E Guze SB. Establishment of diagnostic validity in psychiatric illness: its application to schizophrenia. Am J Psychiatry. (1970) 126:983–7. 10.1176/ajp.126.7.983 5409569

[29] Guze SB. Biological psychiatry: is there any other kind? Psychol Med. (1989) 19:315–23. 10.1017/S0033291700012356 2762437

[30] Wender PH. The minmal brain dysfunction syndrome in children: I-the syndrome and its relevance for psychiatry, II- a psychological and biochemical model for the syndrome. J Nervous Ment Dis. (1972) 155:55–71. 10.1097/00005053-197207000-00007 4402515

[31] Wender PH. The minimal brain dysfunction syndrome. Annu Rev Med. (1975) 26:45–62. 10.1146/annurev.me.26.020175.000401 1096774

[32] Coleman AM. Oxford Dictionary of Psychology. New York, NY: Oxford University Press (2001).

[33] Reed SK. Cognition Theory and Applications. 6th ed. Belmont CA: Wadsworth/Thomson Learning (2004).

[34] Broadbent DE. A mechanical model for human attention and immediate memory. Psychol Rev. (1957) 64:205. 10.1037/h0047313 13441856

[35] Neisser U. Cognition and Reality: Principles and Implications of Cognitive Psychology. New York, NY: Freeman (1976).

[36] Lerner RM Overton WF. Reduction to absurdity: why epigenetics invalidates all models involving genetic reduction. Hum Dev. (2017) 60:107–23. 10.1159/000477995

[37] Ang B Horowitz M Moncrieff J. Is the chemical imbalance an ‘urban legend'? An exploration of the status of the serotonin theory of depression in the scientific literature. SSM-Mental Health. (2022) 2:100098. 10.1016/j.ssmmh.2022.100098

[38] Edelman GM. Bright Air, Brilliant Fire: On the Matter of the Mind. New York, NY: Basic books (1992).

[39] Edelman GM. Building a picture of the brain. Ann N Y Acad Sci. (1999) 882:68–89; discussion 128–34. 10.1111/j.1749-6632.1999.tb08535.x 10415887

[40] Fuster J. The Prefrontal Cortex: Anatomy, Physiology, and Neuropsychology of the Frontal Lobe. 2nd ed. New York, NY: Raven Press.

[41] Fuster J. Memory and planning: Two temporal perspectives of frontal lobe function. In: Jasper HH, Riggion S, Goldman-Rackic PS, editors. Epilepsey and the Functional Anatomy of the Frontal Lobe. New York: Raven Press (1995). p. 816–9. 7771314

[42] Fuster JM. The cognit: a network model of cortical representation. Int J Psychophysiol. (2006) 60:125–32. 10.1016/j.ijpsycho.2005.12.015 16626831

[43] American Psychiatric Association. Diagnostic and Statistical Manual of Mental Disorders. 4th ed. Washington, DC: American Psychiatric Association (1994).

[44] American Psychiatric Association. Diagnostic and Statistical Manual of Mental Disorders. 5th ed. Washington, DC: American Psychiatric Association (2013).

[45] Campbell SB Szumowski EK Ewing LJ Gluck DS Breaux AM. A multidimensional assessment of parent-identified behavior problem toddlers. J Abnorm Child Psychol. (1982) 10:569–91. 10.1007/BF00920755 7161446

[46] Campbell SB Pierce EW March CL Ewing LJ Szumowski EK. Hard-to-manage preschool boys: symptomatic behavior across contexts and time. Child Dev. (1994) 65:836–51. 10.1111/j.1467-8624.1994.tb00787.x 8045171

[47] Milich R Hartung CM Martin CA Haigler ED. Behavioral disinhibition and underlying processes in adolescents with disruptive behavior disorders. In: Routh DK, editor. Disruptive Behavior Disorders in Childhood. New York, NY: Plenum (1994). p. 109–38.

[48] Schweitzer JB Sulzer-Azaroff B. Self-control in boys with attention deficit hyperactivity disorder: effects of added stimulation and time. J Child Psychol Psychiatry. (1995) 36:671–86. 10.1111/j.1469-7610.1995.tb02321.x 7650090

[49] Bronowski J. Human and animal languages. In: Ariotti PE, editor. A Sense of the Future. Cambridge Massachusetts: MIT Press (1977). p. 104–31.

[50] Faraone SV Biederman J. Neurobiology of attention-deficit hyperactivity disorder. Biol Psychiatry. (1998) 44:951–8. 10.1016/S0006-3223(98)00240-6 9821559

[51] Biederman JJ Faraone SV. Attention-deficit hyperactivity disorder. Lancet. (2005) 366:237–48. 10.1016/S0140-6736(05)66915-2 16023516

[52] Barkley RA Cook E Diamond A Zametkin A Tharpar A Teeter A. International consensus statement on ADHD. January 2002. Clin Child Fam Psychol Rev. (2002) 5:89–111. 10.1023/a:1017494719205 12093014

[53] Thome J Ehlis A-C Fallgatter AJ Krauel K Lange KW Riederer P. Biomarkers for attention-deficit/hyperactivity disorder (ADHD). A consensus report of the WFSBP task force on biological markers and the World Federation of ADHD. World J Biol Psychiatry. (2012) 13:379–400. 10.3109/15622975.2012.690535 22834452

[54] Biederman JJ Faraone SV. Annual Report 2002: The Johnson Johnson Center for Pediatirc Psychopathology at the Massachusetts General Hospital. (2002). Available online at: https://highline.huffingtonpost.com/miracleindustry/americas-most-admired-lawbreaker/assets/documents/8/biederman-center-2002-annual-report.pdf (accessed December 1, 2022).

